# Fine-Mapping an Association of *FSHR* with Preterm Birth in a Finnish Population

**DOI:** 10.1371/journal.pone.0078032

**Published:** 2013-10-29

**Authors:** Sung Chun, Jevon Plunkett, Kari Teramo, Louis J. Muglia, Justin C. Fay

**Affiliations:** 1 Computational and Systems Biology Program, Washington University, St. Louis, Missouri, United States of America; 2 Program in Human and Statistical Genetics, Washington University, St. Louis, Missouri, United States of America; 3 Department of Obstetrics and Gynecology, Helsinki University Central Hospital, Helsinki, Finland; 4 Center for Prevention of Preterm Birth, Cincinnati Children’s Hospital Medical Center, Cincinnati, Ohio, United States of America; 5 Department of Genetics and Center for Genome Sciences and Systems Biology, Washington University, St. Louis, Missouri, United States of America; Vanderbilt University, United States of America

## Abstract

Preterm birth is a complex disorder defined by gestations of less than 37 weeks. While preterm birth is estimated to have a significant genetic component, relative few genes have been associated with preterm birth. Polymorphism in one such gene, *follicle-stimulating hormone receptor* (*FSHR*), has been associated with preterm birth in Finnish and African American mothers but not other populations. To refine the genetic association of *FSHR* with preterm birth we conducted a fine-mapping study at the *FSHR* locus in a Finnish cohort. We sequenced a total of 44 kb, including protein-coding and conserved non-coding regions, in 127 preterm and 135 term mothers. Overall, we identified 288 single nucleotide variants and 65 insertion/deletions of 1–2 bp across all subjects. While no common SNPs in protein-coding regions were associated with preterm birth, including one previously associated with timing of fertilization, multiple SNPs spanning the first and second intron showed the strongest associations. Analysis of the associated SNPs revealed that they form both a protective (OR = 0.50, 95% CI = 0.25–0.93) as well as a risk (OR = 1.89, 95% CI = 1.08–3.39) haplotype with independent effects. In these haplotypes, two SNPs, rs12052281 and rs72822025, were predicted to disrupt ZEB1 and ELF3 transcription factor binding sites, respectively. Our results show that multiple haplotypes at *FSHR* are associated with preterm birth and we discuss the frequency and structure of these haplotypes outside of the Finnish population as a potential explanation for the absence of *FSHR* associations in some populations.

## Introduction

Preterm birth is a complex disorder affecting 12% of pregnancies in the US [1]. While the causes of spontaneous preterm labor are not well understood, multiple lines of evidence indicate a significant genetic contribution. Twin studies estimate that the length of gestation has a maternal heritability of 15–40% [2]–[5]. Prior history of post-term as well as preterm delivery is a strong predictor of the length of gestation in a subsequent pregnancy [6], [7]. Preterm birth also clusters among siblings [6], [8], across generations in kinships [9] and by race [10], [11].

Despite significant genetic effects, genes associated with risk of preterm birth have been difficult to identify. The majority of genetic association studies have involved targeted candidate genes and, while some associations have been found, replicated associations are rare [12]. Currently, genome-wide association studies are underway [13] and there have already been new reported associations [14]. However, replication in independent cohorts is challenging as population structure related to different racial groups can lead to false positives in an initial screen and false negatives in a replication study. Indeed, the length of normal gestation as well as the rate of preterm birth differs across races [10], [11], [15].

Previous work identified follicle-stimulating hormone receptor (*FSHR*) as a candidate gene for preterm birth based on an accelerated rate of evolution in humans compared to other mammals [16]. While polymorphism at *FSHR* was associated with preterm birth in a Finnish as well as an African American population, no associations were found in either a European American or Hispanic American population [16]. The lack of associations in these latter two groups could be a consequence of genetic differences between these populations, either because of the frequency of the risk variant or its interaction with genetic background. However, such heterogeneous associations might also reflect a false positive association. Delineating what effects *FSHR* might have on preterm birth and in what populations relies in part on knowing which variants might alter *FSHR* function or, at the least, those variants most strongly associated with preterm birth.

In females, *FSHR* is required for ovarian and follicular development and loss of *FSHR* causes infertility [17]. While *FSHR* is primarily expressed in the ovary [18], it may also have functions in the uterus or cervix [19]–[22] that could influence the initiation of labor. However, *FSHR* may also influence the length of gestation by its effects on the quality of implantation, which is associated with a number of adverse pregnancy outcomes including preterm labor [23]. Another possibility is that *FSHR* influences the timing of fertilization. A common SNP in *FSHR* (S680N) is associated with time to ovulation and length of the menstrual cycle [24]. Because the length of gestation is measured from the date of birth to an estimated date of fertilization based on the last menstrual period combined with ultrasound, the timing of fertilization could lead to the appearance of slightly altered gestation lengths.

To fine-map the association of *FSHR* with preterm birth and identify potentially functional variants, we sequenced coding and conserved non-coding regions in Finnish preterm and term mothers. We find that neither common nonsynonymous SNPs, including one associated with altered menstrual cycles, nor the aggregate burden of rare variants is associated with preterm birth. The strongest associations are concentrated in introns 1 and 2 and haplotype analysis of this region revealed both a risk and protective haplotype with statistically independent effects. Our analysis of these data reveals both candidate functional variants as well as insight into the population heterogeneity in associations between *FSHR* and preterm birth.

## Results

### Identification of Genetic Variants in *FSHR* Coding and Non-coding Regions

To identify potentially causal variants underlying the genetic association of *FSHR* with preterm birth, 67 candidate regions were sequenced in 127 preterm and 135 term Finnish mothers (Materials and Methods). The sequenced regions cover a total of 17 kb of sequence within candidate regions and an additional 27 kb of sequence flanking the candidate regions. The candidate regions include *FSHR* protein-coding regions as well as any non-coding regions likely to be functional. Non-coding regions were selected based on experimental evidence from the literature, sequence conservation across placental mammals using PhastCons [25], or rapid evolution along the human lineage [16]. Rapidly evolved sequences are of particular interest since they may have influenced changes in the length of gestation during human evolution [16].

**Figure 1:**
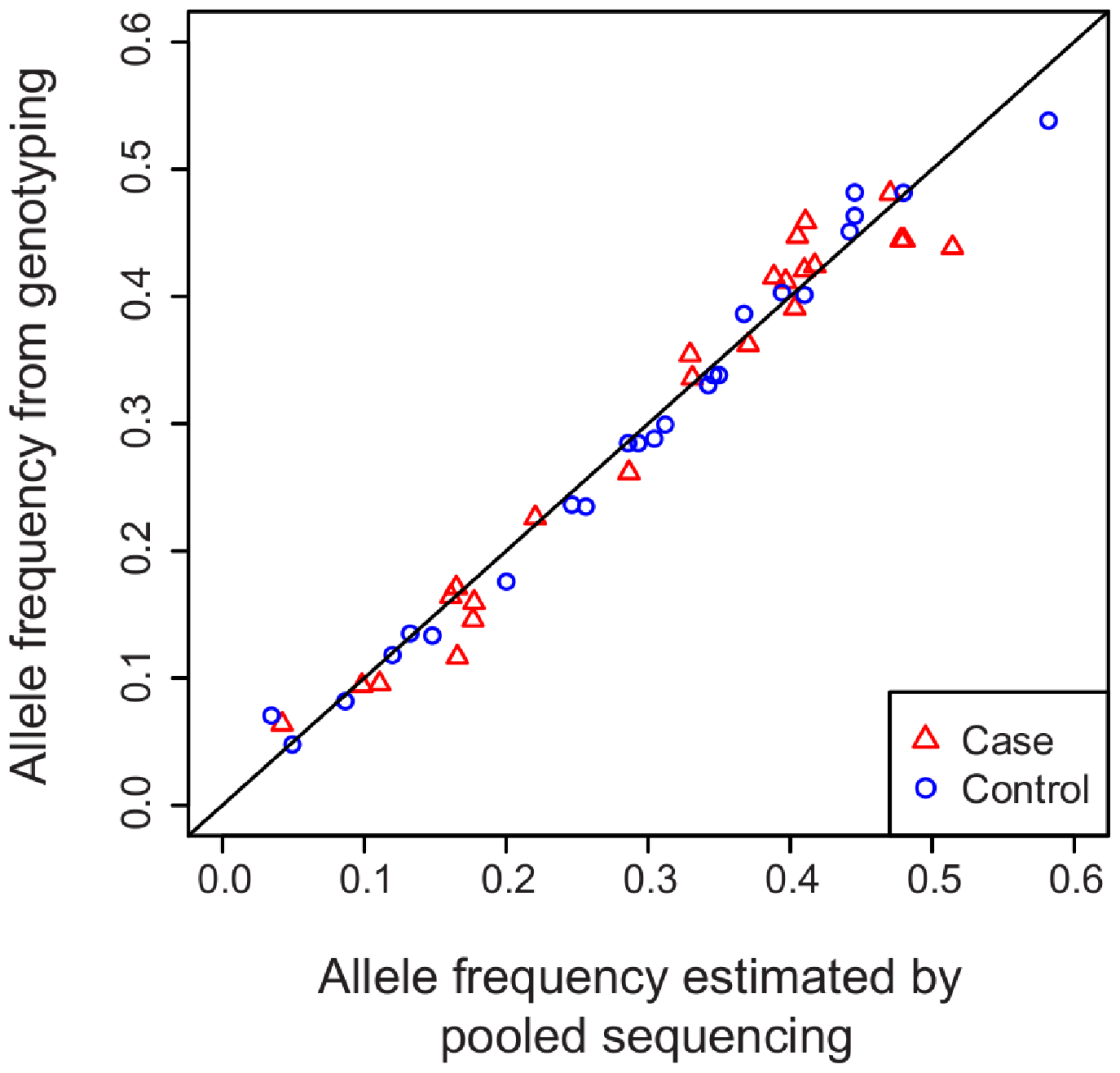
Allele frequencies estimated from pooled sequencing in comparison to Affymetrix genotyping. Allele frequencies for cases (red triangles) and controls (blue circles) are shown for 24 SNPs shared between the two assays. The Affymetrix genotype data are from [16].

Using next-generation sequencing technology, we applied a pooled high-throughput sequencing protocol [26] to sequence the target regions in pools of cases and controls. In total we identified 281 high-quality variants and an additional 72 lower-quality variants (Table 1). The lower quality of some variants can be attributed to either their low frequencies or low read coverage. Nevertheless, we found high power to detect rare variants (see Methods) and accurate estimates of allele frequencies in comparison to those obtained by genotyping (Pearson’s correlation coefficient = 0.99) (Figure 1).

**Table 1:** Summary statistics of sequencing and identified genetic variants.

				High-quality SNVs^1^			Low-quality SNVs^1^		
Pool	Alleles (2N)	Reads	Coverage^2^	SNV	InDel	All^3^	SNV	InDel	All^3^
Case #1	192	34,940,358	74.7	198	42	240 (30)	29	16	45 (44)
Case #2	62	30,017,566	198.7	191	38	229 (60)	19	16	35 (34)
Control #1	150	32,513,156	89.0	202	41	243 (38)	30	13	43 (42)
Control #2	120	35,190,219	120.4	197	36	233 (33)	27	16	43 (43)
Total	524	132,661,299	103.9	236	45	281	52	20	72

1 The number includes single nucleotide variants found in flanking regions as well as in candidate regions.

2 Reads per site per allele.

3 In parenthesis is the number of variants with the estimated allele count less than or equal to 2 in a pool.

### Genetic Variants Altering Protein Sequence

We did not find any frame-shift, splice-site, or nonsense variants in *FSHR*. However, four nonynonymous variants were identified (Table 2), all of which were previously reported in dbSNP and had high variant quality scores in our data. Among them, two alleles, S680N and A307T, were common enough in frequency to be tested for association with preterm birth. In particular, S680N is likely functional since it was previously found to be associated with the length of the menstrual cycle [24] and response to FSH treatment [27]. However, neither S680N or A307T showed significant association with preterm birth (Methods, two-proportion Z-test, P = 1.0 and 0.86, respectively).

**Table 2:** Nonsynonymous SNVs identified in subjects.

			Minor allele frequency			
Variant	Amino acidchange	Functional Prediction^1^	Case	Control	OR	P allelic^2^
rs6166	S680N	Neutral	0.480	0.480	1.00	1.00
rs6165	A307T	Neutral	0.502	0.512	0.98	0.86
rs121909658	A189V	Deleterious	0.004	0.000	n.a.	not tested
rs111883853	R162K	Marginal	0.013	0.026	0.50	not tested

1 Functional predictions were made using a likelihood ratio test.

2 P-values were calculated using a Z test.

We observed two potentially functional nonsynonymous rare variants, A189V and R162K in our samples. A189V was detected at an allele frequency of 0.4% in the case pool and so is likely to be heterozygous in a single individual. The A189V change disrupts an evolutionarily conserved amino acid (P<10^−8^ by the likelihood ratio test [28] and probably damaging by PolyPhen-2 [29]) and is a well-characterized inactivating mutation previously reported to cause ovarian failure in homozygotes [30] and in a compound heterozygote with A419T [31]. Another variant R162K is two-fold over-represented in controls (2.6%) relative to cases (1.3%), although the difference is not significant. Although PolyPhen-2 predicts R162K as benign, the mutated residue is marginally conserved across mammals according to the likelihood ratio test (P = 0.0017), and despite the biochemical similarity between arginine and lysine, lysine was not observed at the orthologous position of 18 placental mammals.

### No Enrichment of Rare Variants in Cases

Although an individual rare variant can explain only a small fraction of the risk for a common genetic disorder, rare variants can still make a substantial aggregate contribution to risk [32], [33]. To test if rare variants in *FSHR* are enriched in preterm mothers, we compared the distribution of rare single nucleotide variants (SNVs), defined by a minor allele frequency less than 1%, between cases and controls. Using either all rare SNVs or rare SNVs at conserved sites, defined by PhyloP (P<0.05) [34], we found no enrichment in cases compared to controls (Table 3).

**Table 3:** Rare SNVs found in candidate regions.

		Case		Control			
Variant quality	Region^1^	Number^2^	Collapsed AF	Number^2^	Collapsed AF	OR^3^	P^4^
high	all	8	3.2%	11	7.0%	0.44	0.03
	conserved sites	5	2.0%	6	4.1%	0.47	0.13
high+low	all	18	6.4%	18	8.6%	0.73	0.21
	conserved sites	6	2.6%	9	4.7%	0.54	0.16
high	flanking controls	13	7.0%	12	6.8%	1.04	n.a.

1 Rare variants were counted in “all” candidate regions or only in “conserved sites” within candidate regions.

2 The rare variants are defined by minor allele frequency below 1% in both case and control groups.

3 Odds ratio of collapsed rare variant frequency in cases relative to controls.

4 One-tail Fisher’s exact test of the collapsed allele frequencies between cases and controls.

To increase the power of the analysis we used several less restrictive sets of rare variants. When the sensitivity of variant detection was maximized at the expense of specificity by lowering the variant quality score cut-offs, a similar number of rare SNVs was found in cases and controls (Table 3). We also found no enrichment of SNVs present exclusively in cases or controls and with minor allele frequencies up to 5% (Table S1). To control for any differences in the sensitivity of detection or sample size we compared SNVs in candidate regions to those within sequences flanking the candidate regions (Table 3 and Table S1). However, flanking sequences showed similar numbers of SNVs in each comparison.

### Fine-mapping the Association of Common Variants

A previous study by Plunkett *et al*. (2011) identified three common tag SNPs, rs11686474, rs11680730 and rs12473815, that were significantly associated with preterm birth with odds ratios of 1.76–1.82 in a Finnish cohort. We observed a slight decline in the odds ratios of these same tag SNPs to 1.58–1.59. The lower odds ratios are due to subtle differences in allele frequency estimates between the two studies as this study only included a subset of subjects from the earlier report. We estimated the minor allele frequency of the three SNPs to be 38.9, 39.7 and 41.7% in cases and 28.6, 29.3 and 31.2% in controls whereas the previous study reported a frequency of 41.6, 41.2 and 42.5% in cases and 28.5, 28.5 and 29.9% in controls.

**Figure 2:**
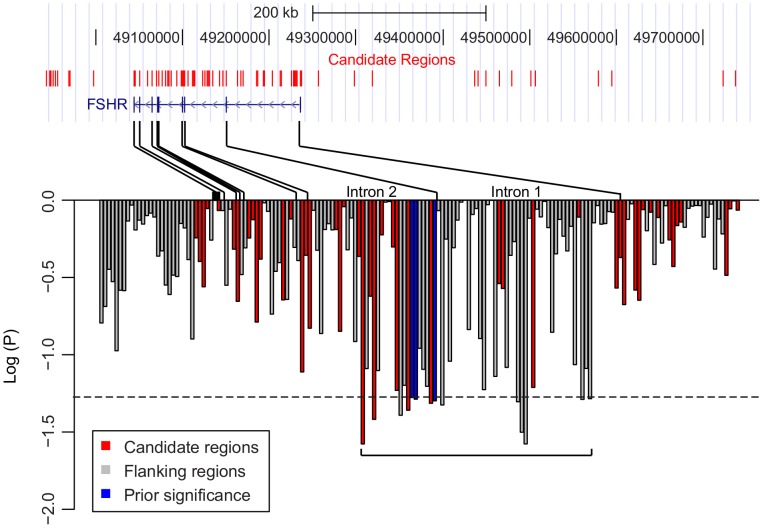
Fine-map of the association between common variants and preterm birth. Each of 169 variants with minor allele frequency greater than 5% was tested for the allelic association with pre-term birth using a two-proportion Z-test. The log_10_ of the P-value is shown for each SNP ordered by is position at the *FSHR* locus, shown above, with lines indicating intron boundaries and red ticks indicating the positions of 67 candidate regions sequenced. Bars are red for SNPs within candidate regions, gray for SNPs in flanking regions, and blue for those found significant in [16]. The dashed horizontal line indicates the least significant P-value of the three tag SNPs in blue.

To fine-map the association of *FSHR* with preterm birth, 169 common variants in sequenced regions were tested for allelic association (Figure 2). The common variants were defined by a minor allele frequency >5% and included 17 InDels and 152 SNPs. The candidate regions included 17 kb of all sequenced regions (39%) and harbored 52 of the common variants (30.8%). We reasoned that a potentially causal variant would exhibit an association at least as strong as the three tag SNPs that were previously identified. Out of 169 tested variants, 11 SNPs had association stronger than the tag SNPs (two proportion Z-test, P<0.05). All 11 associated SNPs were non-coding and localized to a 103 kb region spanning intron 1 and 2 of *FSHR*. The SNP with the highest association (rs12052281) was located in a conserved non-coding element in intron 2 (two-proportion Z-test, P = 0.026).

**Figure 3:**
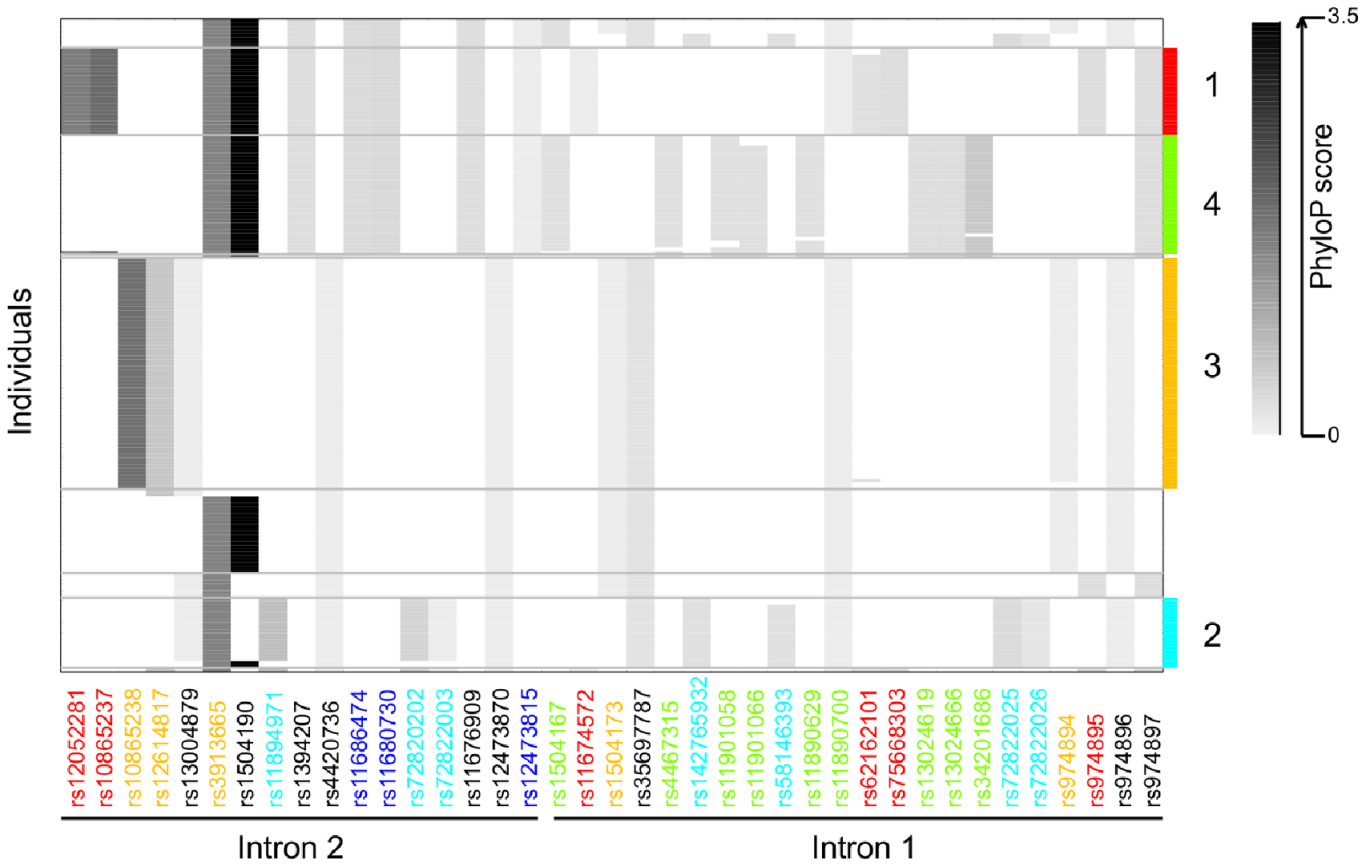
Haplotype structure of common SNPs within the fine-mapped interval. Each row corresponds to a phased chromosome from 93 Finnish individuals in the 1000 Genomes Project. Each column represents one of the 39 SNPs that are segregating in the sequenced regions and also in the fine-mapped interval spanning intron 1 and 2. Derived alleles at conserved sites are shown in gray scale with black being the most highly conserved sites by PhyloP and white showing the ancestral allele. The four major haplotypes are numbered and color coded on the right such that SNPs carried exclusively by one of the four haplotypes have the same label color on the bottom. The three tag SNPs evaluated in [16] are labeled in dark blue.

To explore linkage disequilibrium within the fine-mapped interval bounded by the 11 associated SNPs, we examined the haplotypes of 93 normal Finns (FIN) from the 1000 Genomes Project Consortium (Figure 3, Methods). Within a total of 9 kb sequenced inside the 103 kb fine-mapped interval, 39 SNPs occurred at a minor allele frequency over 5% and clustered into four major haplotypes in FIN. These four haplotypes constitute 77% of chromosomes in FIN; the rest were either rare haplotypes below 5% frequency or could not be directly tagged by a known allele within the sequenced regions. All of the four haplotypes are in linkage disequilibrium with the three tag SNPs that were originally discovered to be associated with preterm birth (D′ = 1.0) [16], but they have low r^2^ (0.28, 0.06, 0.32 and 0.38, respectively).

**Figure 4:**
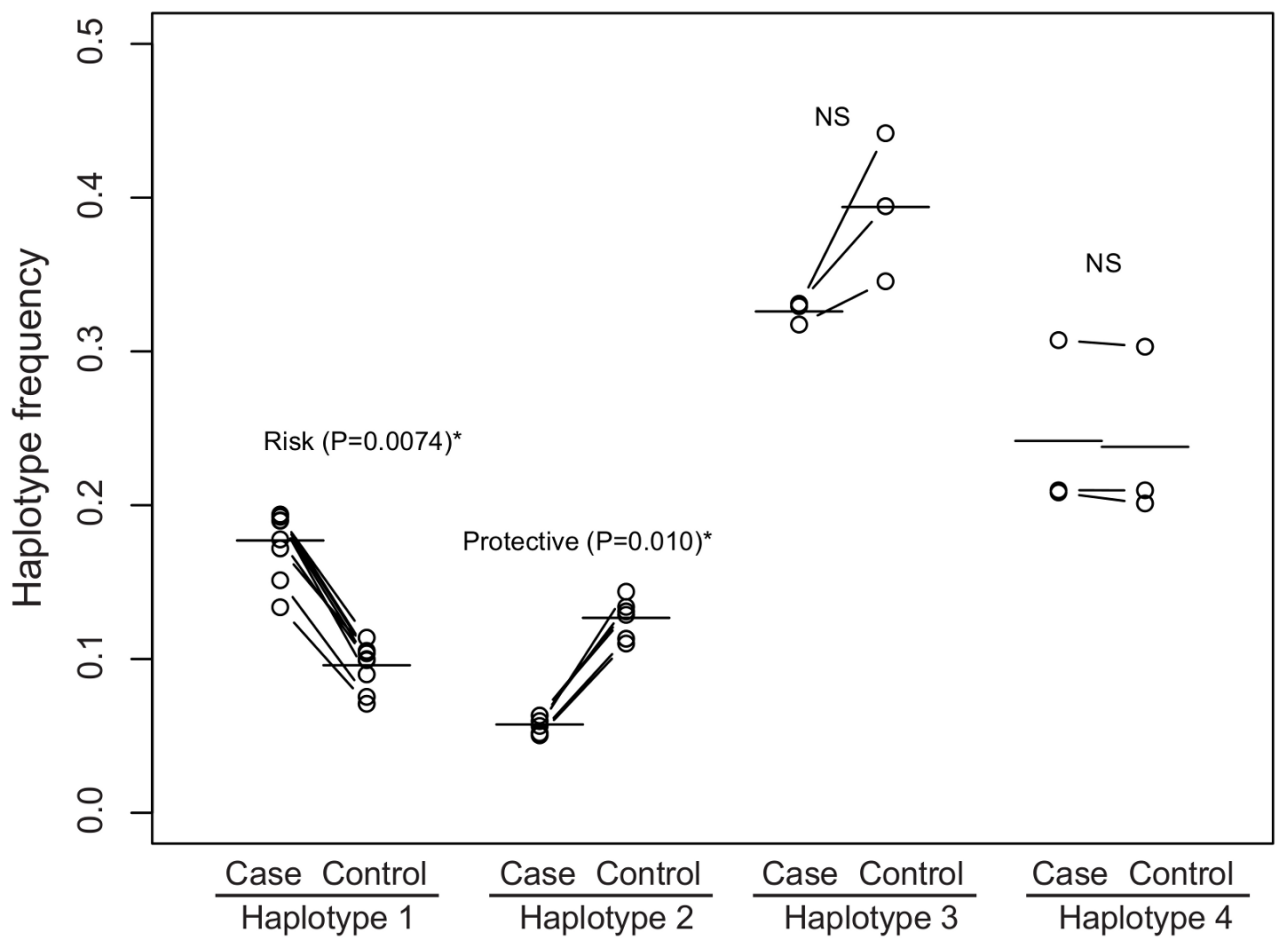
Haplotype frequencies in case and control pools. The haplotype frequencies were estimated from allele frequencies of tag SNPs with r^2^>0.9 (N = 9, 6, 3 and 3 for haplotype 1, 2, 3 and 4, respectively). The horizontal line indicates the average allele frequency among tag SNPs. The difference in allele frequency between cases and controls was tested by Fisher’s Exact Test. NS denotes “not significant.”

To estimate the frequencies of these haplotypes in cases and controls, we utilized 21 SNPs tagging one of the four haplotypes with r^2^>0.9 in FIN (Figure 4, Methods). Based on the genetic homogeneity of Finns, we assumed that the linkage disequilibrium was consistent across FIN and our Finnish preterm and term cohorts. Haplotype 1 showed a significant risk promoting effect (Fisher’s exact test, P = 0.0074, OR = 2.03, 95% CI = 1.17–3.53), and haplotype 2 showed a significant protective effect (P = 0.010, OR = 0.42, 95% CI = 0.21–0.85). The frequencies of haplotype 3 and 4 were not significantly different between cases and controls. To test if the effects of haplotype 1 and 2 are independent from each other, we re-examined the association of haplotype 1 with preterm birth after excluding haplotype 2 from the gene pool, and vice versa. The odds ratios for both haplotypes (OR = 1.87 and 0.48, respectively) remained similar after the correction and were significantly different from 1.0 (Fisher’s exact test, P = 0.020 and 0.023, respectively). The three tag SNPs previously used by Plunkett *et al*. (2011) captured both haplotype 1 and 2 simultaneously, with one allele tagging the risk promoting haplotype and the other tagging the protective haplotype.

To validate the accuracy of the estimated haplotype frequencies we genotyped a set of 12 SNPs across all the samples. Consistent with the haplotype frequencies estimated by sequencing, SNPs tagging haplotypes 1 and 2 were associated with preterm birth (Table S2). Excluding nine individuals with recombinant haplotypes, both haplotypes were significant (Generalized linear model, P<0.05), with an odds ratio of 1.89 (CI = 1.08–3.39, P = 0.0131) for haplotype 1 and 0.50 (CI = 0.25–0.93, P = 0.0343) for haplotype 2. Retaining the nine individuals and conducting the test on each of the three risk and three protective SNPs produced similar results (Table S2).

### Candidate Causal Variants in Risk and Protective Haplotypes

A potential causal risk variant is likely to be exclusively carried by the risk promoting haplotype and not by haplotypes for which we found protective or no effects. In the risk promoting haplotype, six variants satisfying this criterion were found in the 9-kb of sequenced regions within the fine-mapping interval (Figure 3). Note that one of these variants (rs62162101) was moved to chromosome 5 in the latest version of the human genome (NCBI 37) and so was eliminated from our analysis. For each variant, we examined evolutionary conservation at the site, disruption of potential transcription factor binding sites, and functional sequences defined by ENCODE (Methods). Out of the five candidate variants, we identified two in conserved non-coding elements defined by PhastCons, and only one out of the two, rs12052281, was found at a conserved nucleotide defined by PhyloP (P<0.05). None of the variants occurred within potential regulatory sequences defined by ENCODE.

The risk allele of rs12052281 (G) was a derived allele and predicted to impact the binding of the transcriptional repressor ZEB1 by 12.5% (Methods) [35]. A previous study identified ZEB1 as a key suppressor of genes involved in uterine contraction in both humans and mice [36]. FSH, the ligand recognized by FSHR, is known to modulate the electrical signaling property of the uterine muscle [20]. Thus, the weaker binding of ZEB1 in the presence of the G allele may cause de-repression of *FSHR.*

Similarly, the protective haplotype contained seven variants which were not shared with other haplotypes of risk or no effect (Figure 3). Although none of those variants was located within potential regulatory sequences defined by ENCODE or sequence elements conserved across placental mammals, rs72827283 and rs72822025 were within noncoding element founds to be conserved across primates by PhastCon. In particular, the rs72822025 A allele of the protective haplotype occurred within a predicted *ELF3* binding motif defined by a protein-binding microarray and is predicted to reduce the binding energy by 27.1% [37]. In mice, *Elf3* is up-regulated during late pregnancy and activates the prostaglandin synthesis pathway in the uterus through transcriptional activation of *COX-1* [38].

## Discussion

To refine a previously reported association between *FSHR* and preterm birth, we conducted a sequencing-based fine-mapping study in a Finnish population. We ruled out variation in the protein-coding region and found independent associations with both a risk promoting and a protective haplotype that span a ∼100 kb region of intron 1 and 2 of *FSHR*. While we found potentially functional binding site variants within both of these haplotypes, our fine-mapping resolution was limited by high levels of linkage disequilibrium across the region within the Finnish population. As discussed below, our results provide insight into the heterogeneous associations previously observed between *FSHR* and preterm birth [16].

### Rare Variants

One advantage of sequencing over genotyping is the ability to identify rare, potentially functional variants in patient samples. In *FSHR*, there are a number of rare mutations known to cause reduced fertility or ovarian hyperstimulation syndrome [39], which may be risk factors for preterm birth, e.g. [40], [41]. In this study, we found few rare variants within coding or conserved noncoding regions and no enrichment of these rare variants in preterm birth cases compared to controls. Three of the rare SNVs altering nonsynonymous or conserved non-coding sites were only found in preterm mothers and had a combined frequency of 3.6% (Table S3). One of the variants (A189V) was previously associated with ovarian failure [30], [31]. One of the non-coding variants is located at chr2∶49,202,765 in a putative binding site for peroxisome proliferator-activated receptor alpha (PPARA), which has been implicated with preterm birth complicated with infection [42]. The other non-coding variant with MAF of 2.4% is located at chr2∶49,202,863 in a putative binding site for SOX transcription factors and SOX4 was observed to be differentially regulated in the uterus of mice and to a lesser degree in humans [38].

### Common Variants

Within the 796 kb region of the *FSHR* locus that was surveyed, we found the strongest associations within a 103 kb region within intron 1 and 2. This region does not include a common SNP (S680N) previously associated with time to ovulation, length of the menstrual cycle [24], and response to FSH treatment [27]. Because this SNP showed no enrichment in cases compared to controls we can exclude the possibility that it influenced the estimated length of gestation by altering the timing of fertilization.

Within the 103 kb region, we found high levels of linkage disequilibrium and two low frequency haplotypes associated with preterm birth; one with a risk promoting effect (MAF = 12.6%, OR = 1.89) and the other with a protective effect (MAF = 9.1%, OR = 0.50). We validated the independent effects of these haplotypes by individually genotyping a subset of SNPs marking these haplotypes and conducting a combined analysis. While the high level of linkage disequilibrium made it difficult to resolve the association to a smaller region of interest, we identified a number of variants of interest through a bioninformatics analysis of transcription factor binding sites.

Each of the two associated haplotypes has variants that alter potential transcription factor binding sites present within conserved noncoding regions. Two of the variants are noteworthy. The risk promoting haplotype has a weaker ZEB1 binding site and ZEB1 is involved in maintaining uterine quiescence through progesterone receptor activation [36]. The protective haplotype has a weaker ELF3 binding site and ELF3 increases in expression during pregnancy in mice, activating prostaglandin biosynthesis [38]. However, we cannot rule out the possibility of other potentially functional variants outside of our sequenced regions, which included only ∼16% of the fine-mapped interval of intron 1 and 2. Although conserved intronic and intergenic regions in *FSHR* are known to be enriched with *cis*-regulatory elements [43], [44], non-conserved regions can contain transposable element insertions which can modulate gene expression.

### What can Explain Population-specific Associations between *FSHR* and Preterm Birth?

*FSHR* was previously found associated with preterm birth in a Finnish and African American population but not in a European American or Hispanic American population [16]. One potential explanation for this observation is that the causative allele(s) have different frequencies in different populations. Consistent with this possibility, the protective haplotype is at much lower frequency outside of the Finnish population. As ascertained by the 1000 Genomes Project [45], the three SNPs that we used to tag the protective haplotype (rs12465603, rs17038097, rs72822025) are at lower frequency in other European (CEU: 2.9–4.1%, GBR: 2.8%, IBS: 0%, TSI: 2.6%) and African (ASW: 1.6%, YRI: 0%, LWK: 0.5–3.1%) populations compared to the Finnish population (9.7–13%), which is similar to the frequency of these SNPs in our Finnish control samples (12–14%). Interestingly, the frequency of these SNPs is much higher in Hispanic/American populations (MXL: 22–38%, CLM: 9.1–11%, PUR: 9.1–11%). Of note, the frequency of preterm birth is low in both Finland (5.5%) and Mexico (7.3%) compared to the global average (11.1%) [46]. The three SNPs that we used to tag the risk haplotype (rs10865237, rs12614875, rs1504183) are at similar or higher frequencies in other European (15–16%), African (11–34%) and Hispanic/American (20%) populations compared to the Finnish (14%) and our control samples (9.1–11%). Thus, we can expect the near absence of the protective haplotype in some populations to weaken the power of association with common tag SNPs.

Another factor that could lead to different associations in different populations is linkage disequilibrium. While the three SNPs we used to tag the protective and risk haplotypes have a strong r^2^ in the Finnish, an average of 0.94 and 0.82, respectively, they have much lower correlations in European (0.48) and African (0.66) populations, respectively. The lower r^2^ between SNPs that tag the risk haplotype occurs in other European (r^2^ = 0.78) and Hispanic/American (r^2^ = 0.72) populations, but is most pronounced in African populations (r^2^ = 0.18). The average r^2^ between the three SNPs that tag the risk haplotype and the three common tag SNPs found significant in Plunkett *et al*. (2011) is 0.26 in the Finnish population but 0.17 in other European populations, 0.17 in African populations and 0.28 in Hispanic/American populations. Thus, linkage disequilibrium is reduced in some populations.

In summary, both the reduced frequency of the protective haplotype and reduced levels of linkage disequilibrium provide an explanation for the absence of association between *FSHR* and preterm birth outside of Finnish cohorts. While this observation provides one explanation for the absence of association in European American populations it does not explain the absence of association in Hispanic American populations or the presence of association in African American populations. Both haplotype frequencies and linkage disequilibrium are reduced in African populations but not reduced in Hispanic populations. Thus, while Finnish cohorts are advantageous due to population homogeneity and high levels of linkage disequilibrium, they can be disadvantageous when trying to replicate associations in other populations.

### Conclusions

Using a sequenced-based, fine-mapping approach, we have shown that a previous association between *FSHR* and preterm birth is best explained by a risk promoting and protective haplotype that extend over ∼100 kb of intron 1 and 2. Our work highlights the challenges of fine-mapping linkage disequilibrium-based associations, which is important to understanding heterogeneous associations across populations and the mechanisms by which genetic variants influence phenotypes of interest.

## Materials and Methods

### Candidate Regions

Candidate regions covering a total of 17 kb were selected based on an increased likelihood of containing functional variants. These regions include: all exons (NM_000145), 50-bp exon-intron junctions, core promoter region (−1 to −225 relative to the translational start site) [47], 3 SNPs found significantly associated with preterm birth in African Americans (rs11686474, rs11680730 and rs12473815) [16], 15 non-coding elements rapidly evolved along the human lineage [16], and conserved non-coding elements (Table S4). Conserved non-coding elements, a total of 8.6 kb (N = 269), were identified within the transcribed region and 5 kb upstream and downstream, based on the sequence conservation across 32 placental mammals using PhastCons [25]. A PhastCons element was selected as a candidate if it was longer than 50 bp by itself or a part of a cluster of PhastCons elements separated by less than 200 bp and together span more than 50 bp. In addition to PhastCons regions, we also included conserved functional non-coding elements from the literature. One transcriptional silencer [43] and seven distal transcriptional regulatory elements [44] were previously identified in rat, and their sequences are well conserved to human. The genomic coordinates of the rat non-coding elements were transferred to the human genome with UCSC LiftOver (http://genome.ucsc.edu/cgi-bin/hgLiftOver).

The candidate regions were amplified in 67 PCR amplicons (44 kb). PCR primers were designed using Primer 3 (http://frodo.wi.mit.edu/primer3/input.htm) with a minimum amplicon size of 300 bp and other parameters previously described [48], [49]. To avoid allele-specific PCR failure, PCR primers were selected within polymorphism-free segments utilizing the 1000 Genomes Project data [45].

### Ethics Statement

The study of human subject was approved by Institutional Review Boards and Ethics Committees at Helsinki University Central Hospital and Washington University. Informed consent for the genetics research was obtained in writing.

### Human Subjects

The human subjects investigated in this study largely overlap with the Finnish mothers in which the association of *FSHR* was originally identified [16]. Out of 127 preterm and 135 term mothers investigated in this work, 96 cases and 70 controls were shared with the previous study, and the rest were unique to this study. The inclusion criteria for preterm mothers was a non-atrogenic singleton pregnancy with less than 37 completed weeks of gestation without a sign of trauma, infection, or drug abuse. Controls were mothers who delivered spontaneously after 37 gestational weeks, with no other pregnancies delivering preterm or first degree family members born preterm. There was no difference in the average maternal age between cases (30.6 years) and controls (31.4 years) (Wilcoxon test, P = 0.17). The sample genomic DNA was collected from peripheral bloods or saliva using standard methods.

### Pooled High-throughput Sequencing

Human subjects were placed into four groups by case/control status and by whether an individual was also included in a previous study [16] or not. For each group, an Illumina sequencing library was prepared following the pooled high-throughput sequencing protocol [50], [51]. Although this protocol cannot assay individual genotypes, single nucleotide variants (SNVs) and 1–2 bp insertions/deletions (InDels) can be efficiently identified with the high sensitivity and specificity along with their allele frequencies [50], [51]. Briefly, genomic DNA samples in each group were fluorescently quantified using SYBR Gold (Invitrogen) staining technique [51], and mixed into an equimolar pool. To average out stochastic noise, we prepared two technical replicates of pooled genomic samples and repeated all of the subsequently described steps.

In each pool, the candidate regions were amplified by PCR with PfuUltra High-Fidelity DNA polymerase (Stratagene) in the presence of 1M betaine (Sigma-Aldrich). In each PCR reaction, 0.3*N* ng of pooled genomic DNA was added as templates, where *N* is the number of pooled individuals. While the number of PCR cycles was fixed to 28, other PCR parameters were optimized for each amplicon (Table S5). All PCR products were purified on QIAquick spin columns (Qiagen), validated on agarose gel and then quantified again by SYBR Gold staining.

To control for the sensitivity and specificity of pooled sequencing, we prepared positive and negative controls as described previously [51]. The negative control was a 1.9 kb region of M13mp18 plasmid (NEB), and positive controls were 335-bp synthetic sequences derived from *TP53* (shared by F. L. M. Vallania). Seven positive control plasmids carrying a total of 13 known mutations were spiked into unmutated plasmid DNA at the lowest allele frequency of each pool (1/2*N*). Both positive and negative controls were PCR-amplified similar to candidate regions.

Amplicons of candidate regions and controls were pooled in an equimolar ratio so that all regions were sequenced at an even read coverage. The pooled amplicons were randomly ligated into >10 kb concatemers and then sonicated into 100–500 bp fragments with a Bioruptor XL (Diagenode) following [50]. From the sonicated fragments, Illumina sequencing libraries were generated using the Genomic DNA Sample Prep Kit (Illumina). Each library was tagged with an index unique to each subject group. To minimize run-specific variation in error rates, all four sequencing libraries were multiplexed and sequenced on a single Illumina HiSeq lane in a single-read 42-cycle mode.

### Identification of Single Nucleotide Variants

For each pool, 42-bp sequence reads were mapped, aligned, calibrated and then scanned for single nucleotide variants (SNVs) and 1–2 bp insertions/deletions (InDels) using SPLINTER (version 6t) [51]. Out of 35.8–40.4 million reads, 30.0–35.2 miilion (84–88%) were aligned to the reference sequence (hg18) allowing two or less edits per read in the alignment. To calibrate sequencing error rates, a second-order SPLINTER error model was generated from the reads aligned to the negative control sequence. Overall, the error rates were substantially higher at 23 sequencing cycles compared to the rest of cycles. After masking out all such error-prone cycles, only high-quality basecalls (19 nt per read) were utilized to identify SNVs.

The power to detect SNVs goes up with an increasing read coverage but saturates above ∼30 reads per site per allele [51]. We obtained 74.7–198.7 reads per site per allele on average for each pool, which is well above the saturation point. However, the read coverage was still less than 30 reads per site per allele at 2.1% of sites, either due to random sampling or difficulty in alignment. These regions are located in two rapidly evolving elements, a rat distal *cis*-regulatory element (ECR6) and a variable poly-dA region (11–17 bp) in the core promoter.

The optimal cut-offs for SNV calls were determined by positive and negative controls spiked into the sequencing library. In all pools, SNV quality scores of positive controls were well separated from those of negative controls; thus there exist a range of score cut-offs discriminating positive from negative controls with 100% accuracy. We defined SNV sets at high and lower variant quality thresholds to maximize the specificity and the sensitivity, respectively. For each pool, the high quality cut-off was defined by the lowest variant quality score of positive controls whereas the lower quality cut-off was defined by the highest variant quality score of negative controls. After excluding a tri-allelic variant, we obtained a total of 281 high-quality SNVs and 72 lower-quality SNVs across all pools. The allele frequency estimates by SPLINTER were rounded off to the nearest multiple of a singleton allele frequency in each pool.

We validated the power and accuracy of variant identification in three ways. First, using spike-in plasmid controls we found that positive controls added at the singleton allele frequency were all recovered with no false positive. Second, a large proportion of SNVs identified in our subjects are variants also present in the dbSNP database (release 135). The known variants were highly enriched among both of the high- and lower-quality SNVs (93.0% and 38.1%, respectively) compared to raw sequence variants which did not satisfy variant detection criteria (1.0%). Third, the estimated allele frequencies were compared to 24 SNPs that were previously genotyped with Affymetrix microarrays in a largely overlapping set of individuals [16].

### Comparison of Allele Frequencies between Cases and Controls

Significant differences in allele frequency between cases and controls were tested for all common variants of minor allele frequency over 5% using a two-proportion Z-test:
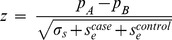


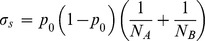


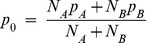
where *p_A_* and *p_B_* are the allele frequencies in cases and controls, respectively, *N_A_* and *N_B_* are the number of alleles in case and control pools, respectively, *p_0_* is the pooled allele frequency over *p_A_* and *p_B_, σs* is the variance component due to random sampling, and the statistic *z* follows the standard normal distribution. Unlike a usual two-proportion Z-test, we had to account for the error of allele frequency estimation as an additional source of variation independent of random sampling. The error of allele frequency estimation *se* was inferred from the mean squared error of observed allele frequencies compared to the expected values using 24 SNPs genotyped in the Finnish cohort with Affymetrix arrays [16]:



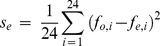
where *f_o,i_* and *f_e,i_* are the observed and expected allele frequencies of SNP *i*, respectively. The *s_e_* was estimated to be 0.00081 for cases and 0.00032 for controls.

### Haplotype Analysis

The haplotype structure of Finnish population was obtained from the 1000 Genomes Project (release 20120316) [45], in which genotypes of 93 individuals (FIN) were phased across the genome using MaCH/Thunder [52]. Ancestral alleles inferred from three sister primate genomes were also obtained from the 1000 Genomes Project. Conserved sites were identified using PhyloP scores across placental mammals [34], which were downloaded from the UCSC genome browser.

The haplotype frequencies were estimated in our case and control subjects by averaging the allele frequencies of multiple tag SNPs, which were selected by tight linkage disequilibrium between the SNP and the haplotype. Specifically, we looked for SNPs with r^2^ greater than 0.9 in Finns from the 1000 Genomes Project (FIN). For haplotype 1, excluding one outlier, nine tag SNPs were utilized to quantify the haplotype frequency. The frequencies of haplotype 2, 3 and 4 were estimated using six, three and three tag SNPs, respectively.

Haplotype frequency was validated by genotyping 12 SNPs across all samples. The 12 SNPs include three that tag the risk haplotype and three that tag the protective haplotype. Individual DNA samples were genotyped for each SNP by Sequenom massARRAY technology at the Human Genetics Division’s Genotyping Core Facility at Washington University. Genotype data are provided in Table S6. Four individuals (1 case and 3 controls) were excluded due to low genotyping quality. The average difference between the pooled allele frequency estimates and the genotype-based frequencies was 1.45% for cases and 0.95% for controls, excluding one marker (rs3913665) for which the minor allele frequency was 31.7% in cases and 34.6% in controls by sequencing and 35.7% in cases and 40.2% in controls by genotyping. Recombinant haplotypes were detected in nine individuals (two cases and seven controls) based on the SNPs used to tag either the protective or risk haplotypes. These nine individuals cause slight differences in P-values and LD of SNPs tagging a haplotype (Table S2). LD relationships and P-values are identical if these nine individuals are removed. A generalized lineage model with a binomial error distribution was used to test for significant effects of both the risk and protective haplotypes.

### Computational Prediction of Transcription Factor Binding Sites

We examined three databases of transcription factor (TF) binding profiles: TRANSFAC [53], JASPAR [54] and UniProbe [55]. TRANSFAC contains the most comprehensive collection of TF binding profiles, JASPAR has fewer but higher-quality profiles, and UniProbe encompasses mostly zinc finger binding motifs, which were derived from *in vitro* protein-binding microarray experiments. In TRANSFAC (version 10.2), the human reference sequences with ancestral alleles were scanned for binding sites of vertebrate TFs using the ECR browser (http://ecrbrowser.dcode.org/) [56]. In JASPAR, the CORE Vertebrata collection of binding matrices was examined using its web server (http://jaspar.cgb.ki.se/) with the default parameters. In UniProbe, human and mouse TFs were scanned with the default setting (http://the_brain.bwh.harvard.edu/uniprobe/), but SNPs with more than 20 hits of predicted binding sites were filtered out for potential non-specificity. For JASPAR and UniProbe, the reference sequences were examined with both derived as well as ancestral alleles in order to explore the gain as well as loss of binding sites. The binding sites predicted with the equivalent score for both ancestral and derived alleles were excluded. The binding energy for ZEB1 and ELF3 was calculated for each allele using ConSite [57] with the default parameter values and published binding site profiles [35], [37].

### Analysis of ENCODE Data

ENCODE data was used to identify potential regulatory SNPs [58]. We classified SNPs as potentially involved in gene regulation if they occurred in open chromatin, defined by DNaseI hypersensitive sites from 125 cell lines or FAIRE, or if they occurred in bound sequences from 194 Chip-seq experiments that make up the ENCODE regulatory track from Yale/UC-Davis/Harvard. Out of the 5 variants unique to the risk haplotype and 7 variants unique to the protective haplotype, none occurred in potential regulatory sequences defined by ENCODE.

## Supporting Information

Table S1 Rare SNVs (<5%) observed in either cases or controls but not both.
(XLS)

Table S2 Genotypes of SNPs tagging protective and risk haplotypes.
(XLS)

Table S3 Rare SNVs exclusively found in cases and located at conserved or nonsynonymous sites.
(XLS)

Table S4 Genomic regions selected for sequencing.
(XLS)

Table S5 PCR parameters used to amplify candidate regions.
(XLS)

Table S6 Genotypes of 12 SNPs across the case/control cohorts.
(XLS)
